# Introduction to the spotlight collection on bioinorganic chemistry

**DOI:** 10.1039/d5sc90092a

**Published:** 2025-05-23

**Authors:** Abhishek Dey, Mi Hee Lim, Serena DeBeer

**Affiliations:** a Indian Association for the Cultivation of Science India icad@iacs.res.in; b Korea Advanced Institute of Science and Technology South Korea miheelim@kaist.ac.kr; c Max Planck Institute for Chemical Energy Conversion Germany serena.debeer@cec.mpg.de

## Abstract

Abhishek Dey, Mi Hee Lim, and Serena Debeer introduce the Royal Society of Chemistry Spotlight Collection on Bioinorganic Chemistry, featuring papers published in *Chemical Science* and *Dalton Transactions*.
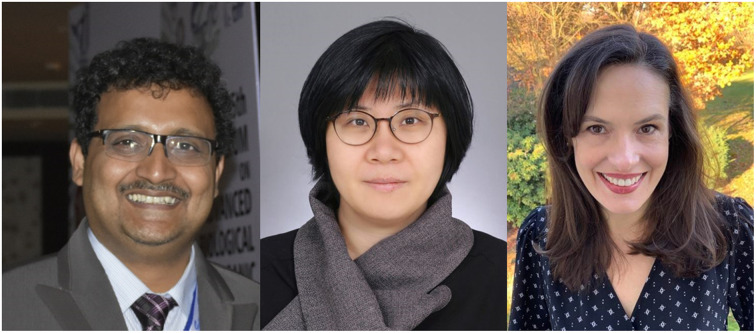

Bioinorganic chemists broadly explore the roles of metal ions in various life processes, applying this knowledge to develop impactful applications, such as metallodrugs, sensors, and catalysts. The fundamental questions driving this field have led to the advancement of numerous synthetic, spectroscopic, analytical and theoretical methods, which have benefitted not only bioinorganic chemists, but also the broader scientific community and industry. Not surprisingly, bioinorganic chemistry has long attracted scientists from diverse backgrounds across the globe, making it one of the most inter-disciplinary and collaborative areas of research. The spotlight collection on “Bioinorganic chemistry”, featuring articles from *Chemical Science* and *Dalton Transactions*, highlights the remarkable diversity inherent in this field.

## Metalloenzyme biochemistry

A central focus of bioinorganic chemistry lies in understanding the biochemistry and biophysical chemistry of metalloenzymes in nature. Behera and co-workers investigate the role of nanoconfinement in ferroxidase activity involved in iron biomineralization within bacterioferritins (https://doi.org/10.1039/D4SC07021F). In a related study, Behera reports enhanced stability of chitosa-functionalized ferritin for iron delivery with minimal cage degradation and leakage (https://doi.org/10.1039/D4DT01839G). Fujii contributes to our understanding of the electronic structure of blue copper proteins through L_3_-edge XANES to reveal that azurin has a more covalent Cu–S bond than amicyanin (https://doi.org/10.1039/D4DT02891K). Elliott examines the reactivity of bacterial cytochrome *c* peroxidase using a combination of spectroscopic techniques to demonstrate that the oxidant produced by the reaction of H_2_O_2_ with the diferric resting state is compound I and not the Fe(iv)Fe(iv)=O species proposed for MauG (https://doi.org/10.1039/D4SC07339H). Dubey employs hybrid QM/MM calculations, MD simulations, and DFT calculations to investigate the mechanism of the C–C bond formation of arylomycin by an engineered cytochrome P450 (https://doi.org/10.1039/D4DT02197E). Ghosh Dey identifies a ferric peroxide (compound 0) intermediate in the peroxidic oxidation of neurotransmitters by heme-bound Aβ peptides relevant to the pathology of Alzheimer's disease (https://doi.org/10.1039/D5DT00234F). Dey uses a combination of self-assembly and *in situ* Raman spectroscopy on naturally-occurring hemes and their artificial analogues to illustrate the interplay between electron withdrawing groups and hydrophobicity in determining spin states and reduction potentials of heme active sites (https://doi.org/10.1039/D5DT00028A).

## Metal-based tools and therapies

Metal-based diagnostic tools and therapeutics are crucial in the detection and treatment of a variety of diseases. Nitric oxide, a key signalling molecule in biology, is the focus of a review by Cho and co-workers, who outline NO donors that can be photo-triggered to release NO on demand under physiological conditions (https://doi.org/10.1039/D4SC06820C). Babak, Ang and co-workers review metal-based inducers of immunogenic cell death and their potential for advancing cancer immunotherapy (https://doi.org/10.1039/D4SC08495K). Zhang and Rahman report that platinum complexes of piperidine-functionalized hydrazone ligands inhibit EZH2-dependent tumorigenesis in pancreatic ductal adenocarcinoma, showing synergistic effects with PARP inhibitors (https://doi.org/10.1039/D4DT01243G). Sasmal presents a biotinylated Pt(iv)–SAHA conjugate for the treatment of cutaneous T-cell lymphoma, designed to enhance the efficacy of cisplatin (https://doi.org/10.1039/D4DT01571A). Hartinger also describes biotin-functionalized Ru(ii)/Os(ii)/Rh(iii)/Ir(iii) hydroquinoline complexes, identifying a Rh complex with high anti-proliferative activity (https://doi.org/10.1039/D4DT02296C). Gasser and Batista introduce two new Ru(ii)–phosphine–mercapto complexes with promising anti-cancer activity (https://doi.org/10.1039/D4DT01191K). Liu, Wu and Chao report a cyclometalated Ir(iii)–tetrazine complex which can generate singlet oxygen in cells *via* two-photon excitation (https://doi.org/10.1039/D4DT01665C). Rudd and Donnelly develop a theranostic strategy for breast cancer by conjugating either β^−^- or β^+^- emitting isotopes, like ^67^Cu or ^64^Cu, to a HER2-targeted sarcophagine-trastuzumab construct (https://doi.org/10.1039/D4SC06969B). For imaging applications, Wilson and co-workers report two pyridine-containing chelators that demonstrate excellent ^111^In binding and delivery in a mouse model (https://doi.org/10.1039/D4DT02146K). Finally, Codd expands the repertoire of metal-binding ligands by designing multimeric linear and macrocyclic hydroxamic acid chelators, which were screened against Ga(iii) or Zr(iv) (https://doi.org/10.1039/D4SC04888A).

## Biomimetic and bio-inspired chemistry

Biomimetic and bio-inspired chemistry is another pillar of bioinorganic chemistry, where researchers strive to replicate the elegant and powerful chemistry of enzymatic active sites using smaller analogues. These analogues can be entirely artificial, as illustrated by Yoo, Choi and Lee's review of nickel-based models of NiCODH (https://doi.org/10.1039/D4SC06957A). Kojima and co-workers develop structurally distinct molecules inspired by CODH that electrochemically reduce CO_2_ to CO (https://doi.org/10.1039/D4DT02922D). Bo, Gil-Sepulcre, Llobet and Shalom report the heterogenization of a molecular ruthenium-based catalyst onto a polymeric carbon nitride photoanode, resulting in a highly efficient water oxidation catalyst (https://doi.org/10.1039/D4SC04678A). Alternatively, naturally-occurring peptides can be harnessed for novel reactivity, as shown by Bren and co-workers, who use a cobalt porphyrin mini-enzyme to reduce CO_2_ (https://doi.org/10.1039/D4SC07026G). Zhang, Chen and Zhang report the oxidation of aromatic rings to phenols using H_2_O_2_ and a binuclear copper catalyst with an asymmetric ligand that mimics the reactivity of tyrosinases (https://doi.org/10.1039/D4DT02872D). Morimoto, Itoh and co-workers explore a series of copper complexes that activate small molecules, such as NO, CO and O_2_, finding that complexes with more positive reduction potentials preferentially bind NO over O_2_ and CO, which emphasizes the importance of ligand design in tuning selectivity (https://doi.org/10.1039/D4DT03001J). Jarjayes, Thomas, Storr and co-workers resonate with this principle in their use of a tetradentate bis-carbene bis-phenolate ligand to stabilize unusual metal nitrides, which undergo M–C insertion reactions (https://doi.org/10.1039/D4DT01765J). The ligand's redox activity aids in lowering the transition-state barrier. Ligand redox chemistry also plays a major role in nickel complexes reported by Ivanović-Burmazović, Goldsmith and co-workers, where a pendant quinol group in the outer coordination sphere enables a non-peptide complex to exhibit promising SOD activity (https://doi.org/10.1039/D4DT03331K). Protein environments also critically influence active site reactivity. Follmer, Borovik and co-workers use a biotin-streptavidin platform to mimic the active sites and reactivity of natural copper metalloenzymes, demonstrating that aromatic residues within the secondary coordination sphere, designed to oxidize substrates with H_2_O_2_, may instead undergo self-oxidation, suggesting a potential deactivation pathway in natural copper enzymes (https://doi.org/10.1039/D4SC06667G). The role of the secondary coordination sphere is further examined by Chen, Rath and co-workers, who use paramagnetic NMR spectroscopy to study substrate binding in synthetic iron porphyrins (https://doi.org/10.1039/D4SC05432F). Berben and co-workers report that including an amine group in the secondary sphere enhances both the selectivity and rate of catalytic hydride transfer reactions. The amine group stabilizes the metal hydride intermediate *via* hydrogen bonding, thereby preventing undesired hydride transfer to CO_2_ and avoiding formate production (https://doi.org/10.1039/D4SC07359B). Similarly, Kovacs shows that hydrogen bonding slows O_2_ binding in a thiolate-bound non-heme iron complex, offering vital insights into how electronic structure controls function in enzymes, such as CDO and IPNS (https://doi.org/10.1039/D4SC02787F).

As bioinorganic chemistry continues to grow in scope, more and more researchers are joining in with new expertise to address unanswered questions that still perplex us. New techniques under development herald the promising future of this community. We look forward to these with excitement. We enjoyed editing this spotlight collection and we hope that the readers enjoy reading these articles too.

